# Eu^3+^-doped Bi_4_Si_3_O_12_ red phosphor for solid state lighting: microwave synthesis, characterization, photoluminescence properties and thermal quenching mechanisms

**DOI:** 10.1038/srep42464

**Published:** 2017-02-15

**Authors:** Yan Zhang, Jiayue Xu, Qingzhi Cui, Bobo Yang

**Affiliations:** 1 School of Materials science and Engineering, Shanghai Institute of Technology, Shanghai 201418, P.R. China

## Abstract

Europium-doped bismuth silicate (Bi_4_Si_3_O_12_) phosphor has been prepared by microwave irradiation method and its crystal structure is determined using Rietveld method. As-prepared phosphor consists of spherical, monodispersed particles with few agglomeration, high crystallinity, and narrow grain size distribution. The phosphor can be efficiently excited in the wavelength range of 260–400 nm, which matched well with the emission wavelengths of NUV LED chips. The photoluminescence spectra exhibit the highest emission peak at 703 nm originating from ^5^*D*_0_ → ^7^*F*_4_ transition of Eu^3+^ under NUV excitation. The luminescence lifetime for Bi_4_Si_3_O_12_: 2 at% Eu^3+^ phosphor decreases from 2.11 to 1.86 ms with increasing temperature from 10 to 498 K. This behavior of decays is discussed in terms of radiative and nonradiative decays dependence on temperature. The thermal quenching mechanism of ^5^*D*_0_ emission of Eu^3+^ in Bi_4_Si_3_O_12_ phosphor is a crossover process from the ^5^D_0_ level of Eu^3+^ to a ligand-to-europium (O^2−^ → Eu^3+^) charge transfer state. The quantum efficiency of the phosphor under 393 nm excitation is found to be 14.5%, which is higher than that of the commercial red phosphors Y_2_O_3_: Eu^3+^, Y_2_O_2_S: Eu^3+^. The temperature effect on CIE coordinate was discussed in order to further investigate the potential applications.

White light-emitting-diodes (WLEDs) as the next-generation general lighting source is extensively studied due to high efficiency, long lifetime, fast response, energy saving and environment friendliness etc^1^,^2^. Presently, the commercial WLEDs comprises a blue LED chip with yellow phosphor YAG: Ce^3+ ^3. However, YAG-based white LEDs suffers from poor color rending index (CRT) (Ra < 80) and high correlated color temperature (CCT > 5000 K) due to the lack of red light component. A combination of near-ultraviolet (NUV) LED chip with tricolor (red, green and blue) (RGB) phosphors or a combination of R/G phosphors with a blue LED chip is another strategy to generate white LEDs. The WLEDs fabricated by this strategy can provide high color rending index (Ra > 90)^4^, appropriate color temperature and high color tolerance to chip’s variation^5^,^6^. The WLEDs based on NUV LED chip coated with tricolor phosphor is not widely used owing to the lack of red phosphors with high conversion efficiency. The commonly used sulphide based red phosphor (Y_2_O_2_S: Eu^3+^) in WLEDs could not efficiently absorb NUV light emitted by NUV LED chip^7^. Therefore, the demand for new red phosphors, which can emit suitable red light for inclusion with the blue LED-YAG: Ce^3+^ combination, is very urgent.

In the search of red emitting phosphors that can be pumped by near-UV or blue emitting LEDs, the nitride based red phosphors and Mn^4+^ -doped activated oxide/fluoride red phosphors emerged as attractive red phosphor for white LED^8^,^9^,^10^,^11^,^12^,^13^. The nitrides (e.g., Ca_2_Si_5_N_8_: Eu^2+ ^14, CaAlSiN_3_: Eu^2+ ^15, (Sr, Ca)AlSiN_3_: Eu^2+ ^16) and oxynitrides (SrSi_2_O_2_N_2_: Eu^2+ ^17) seem to be promising candidates; unfortunately, the high cost caused by the rather demanding synthesis and lower luminous efficacy resulting from broad emission band limit their practical application. Mn^4+^ doped oxide phosphors with broad band emissions exhibit weak absorption for blue excitation light, and Mn^4+^ doped fluoride phosphors are suffering from thermal stability and high humidity degradation^18^. The optimal red-emitting phosphor for a warm-white LED with a high quantum efficiency and high color quality has a narrow emission band (FWHM < 30 nm) located between 615 and 655 nm^19^. Eu^3+^ doped phosphors are probably the better choice because Eu^3+^-doped phosphors exhibit higher luminescence efficiency and stronger red emission compared with other luminous materials, due to the facts that Eu^3+^ (4*f*^6^) ion emits a narrow band, almost monochromatic light and has a long lifetime of the optically active states^20^,^21^,^22^,^23^,^24^,^25^,^26^,^27^.

With good physical, chemical and mechanical properties, bismuth silicate (Bi_4_Si_3_O_12_) is a well-known scintillator and also used as the host for lasers and phosphors^28^,^29^,^30^,^31^. The conventional preparative methods for this phosphor require temperature in the range of 850–1000 °C and several hours soaking time^32^,^33^. Bi_4_Si_3_O_12_ obtained from this method encounters a few problems, such as inhomogeneity, low surface area and broad particle size distribution. In microwave system, since microwave irradiation directly couple microwave energy to the molecules that are present in the reaction mixture, rather than being supplied from the external, uniform and rapid heating can be realized within a short time and at a temperature lower than that normally required. Therefore, compared with the conventional methods, microwave irradiation offers several advantages, including reduced reaction time, small particle size, narrow particle size distribution, better selectivity, and higher reaction yield^34^,^35^. This route leads a mixture at the molecular level of the constituent and better product purity. Different types of nanoparticles such as metal, semiconductors, and oxides have been synthesized using microwave irradiation^36^,^37^,^38^,^39^,^40^,^41^.

In this work we report the preparation of the Eu-doped Bi_4_Si_3_O_12_ phosphors processed by microwave synthesis route. The structure, morphology and particle size distribution of the Bi_4_Si_3_O_12_: Eu phosphors have been studied by means of X-ray diffraction (XRD), scanning electronic microscopy (SEM), and laser particle size analyzer. The temperature dependence of the luminescence properties and the decay time are investigated and discussed for the phosphors. And the thermal quenching mechanisms as well as the quantum efficiency and temperature dependence of the CIE chromaticity of Eu^3+^ in the Bi_4_Si_3_O_12_ matrix are also discussed.

## Results and Discussion

### Structural and morphology characterization

**Figure 1:**
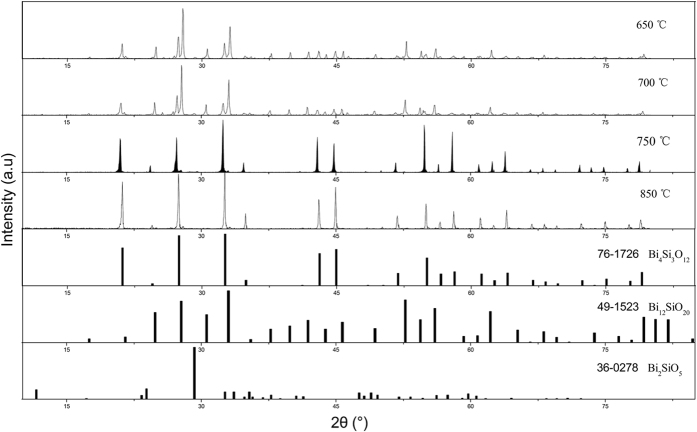
Powder X-ray diffractograms of microwave-prepared Bi_4_Si_3_O_12_: 2 at% Eu^3+^.

The XRD patterns of the as-obtained Bi_4_Si_3_O_12_: 2 at% Eu^3+^ powder obtained at various microwave irradiation temperature are exhibited in Fig. 1. For the samples prepared by the microwave synthesis method, the Bi_12_SiO_20_ and Bi_2_SiO_5_ phases are observed as impurity phases when the calcination temperatures are 650, 700, 800 °C. When the irradiation temperature is 750 °C, a single phase of Bi_4_Si_3_O_12_: 2 at% Eu^3+^ is obtained. And the XRD pattern of the target material is shown in Fig. 1. It indicates that microwave heating provides satisfactory conditions for the formation of single phase Bi_4_Si_3_O_12_: 2 at% Eu^3+^ in a short time of 900 s at a low calcined temperature of 750 °C.

**Figure 2:**
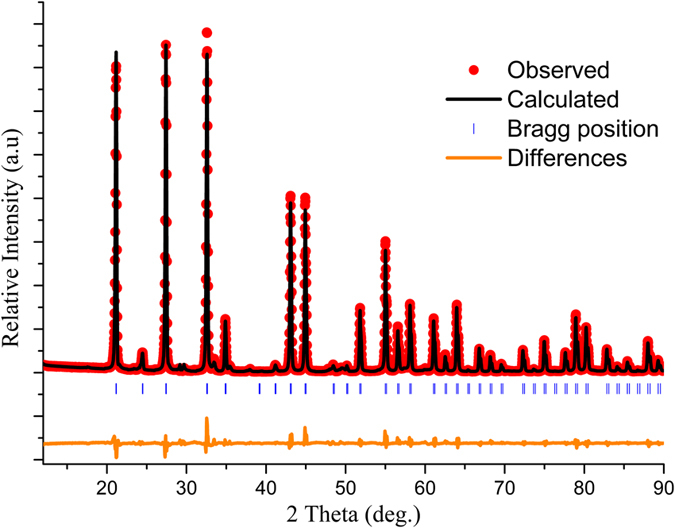
Rietveld refinement of the Bi_4_Si_3_O_12_: 2 at% Eu^3+^ phosphor.

**Figure 3:**
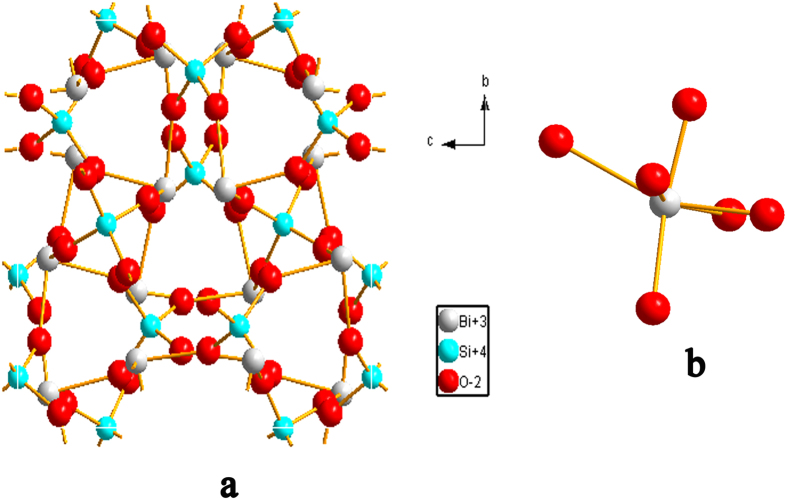
(**a**) Ideal unit cell crystal structure of the Bi_4_Si_3_O_12_: 2 at% Eu^3+^ phosphor, (**b**) coordination of Bi ions doped with Eu^3+^.

The Rietveld refinement is accomplished to obtain the detailed crystal structure information on Eu^3+^-doped Bi_4_Si_3_O_12_ phosphor. The single crystal structure data of Bi_4_Si_3_O_12_ (ICSD No. 84519) are used as the starting structure model for the refinement. Figure 2 shows the observed, calculated, and difference XRD patterns for the Rietveld refinement of Bi_4_Si_3_O_12_: 2at% Eu^3+^ phosphor. The reliability factors finally converges to goodness of fit parameters *χ*^2^ = 4.17%, *R*_wp_ = 9.84% and *R*_p_ = 7.10%, respectively, which shows the validity of the refinement process. The refinement results indicate that Bi_4_Si_3_O_12_: 2at% Eu^3+^ phosphor is related to eulytine structure, and it has I-43d space group with unit cell parameters *a* = *b* = *c* = 10.2831 Å, *V* = 1087.3625 Å^3^, and *Z* = 4. The crystallographic data and atom coordinate are summarized in Table 1. Figure 3 depicts the unit cell structure of Bi_4_Si_3_O_12_ viewing along the *a*-direction and the coordination environment of cation sites.

According to ICSD No. 84519, pure Bi_4_Si_3_O_12_ has a cubic structure with the I-43d space group, and its lattice parameters are *a* = *b* = *c* = 10.2889 Å. With Eu^3+^ incorporation into the Bi_4_Si_3_O_12_ lattice, the XRD peaks slightly shift towards high 2θ range, implying the contraction of the lattice constants. Taking valence states, crystallochemical behavior and ionic radii of Bi^3+^ (117 pm), Si^4+^ (40 pm) and Eu^3+^ (108.7 pm) in consideration, Eu^3+^ ions replaced Bi^3+^ ions site easily. Thus, the substitution of Bi^3+^ ions with Eu^3+^ causes a decrease in the unit cell parameters in view of the fact that the ionic radii of Eu^3+^ (108.7 pm) is slightly smaller than that of Bi^3+^ (117 pm).

**Figure 4:**
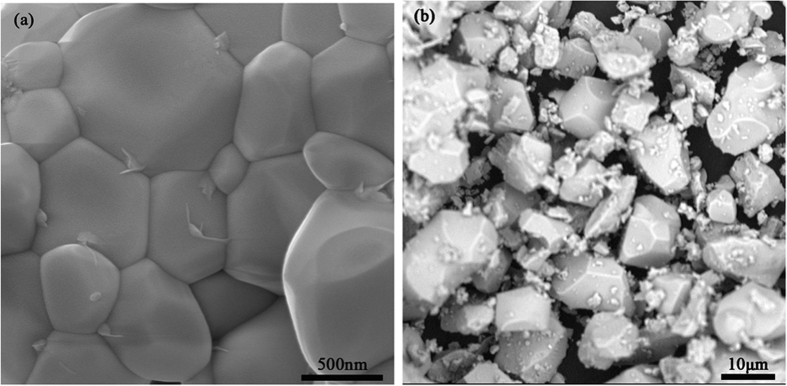
SEM images of Bi_4_Si_3_O_12_: 2 at% Eu^3+^ phosphors synthesized by (**a**) microwave and (**b**) conventional heating.

Figure 4 shows the SEM images of Bi_4_Si_3_O_12_: 2 at% Eu^3+^ phosphors prepared with microwave heating method and conventional solid state reaction method. In the case of the microwave heating method, the synthesized samples consisted of nearly spherical clusters of 0.6–0.8 μm in diameter. The results indicate that the microwave processing can effectively control the particle size and prevent heavy agglomeration, and thus is favorable to synthesis of fine particles of phosphors. On the other hand, when the conventional method was used, the particles aggregated to be consist of tightly packed smaller particles with size about 1–13 μm. This non-uniform particle size is caused due to the non-uniform distribution of temperature and mass flow during the synthesis. In addition, no sintering behavior found in the microwave heating may be attributed to rapid and uniform heating in a quite short time. Nanopowders with homogeneous grain size are an attractive feature for optical applications. In fact, it is well-known that the luminescence characteristics of the phosphor depend on the particle size^42^.

**Figure 5:**
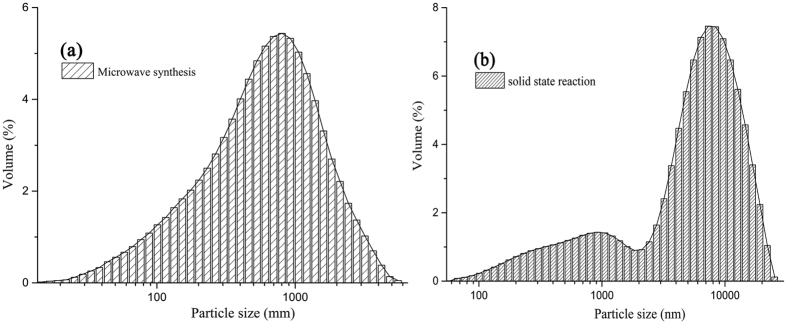
Particle size distribution histograms of Bi_4_Si_3_O_12_: 2 at% Eu^3+^ phosphor synthesized by (**a**) microwave heating and (**b**) conventional solid-state reaction methods.

The size distribution of the Bi_4_Si_3_O_12_:2at% Eu^3+^ particle synthesized by microwave heating and solid state reaction methods is illustrated in Fig. 5. The single peak of particle size distribution is found for the phosphor. The sample exhibits a rather narrow size distribution concentrated in the regions of 694 nm–915 nm (average size 797 nm) with few aggregation or agglomeration. The conventional heating produces a broader particle size distribution of 1 to 10 μm (average size 7 μm). Compared with traditional high temperature solid state reaction, the microwave synthesis method offers advantage of being shorter reaction time for sample preparation. Therefore, the growth of grain size due to the rapid heating is prohibited in the reaction, and pure and single phase Bi_4_Si_3_O_12_: Eu^3+^ particles with finer and uniform microstructure can be obtained by microwave irradiation method.

### Excitation and emission spectra

**Figure 6:**
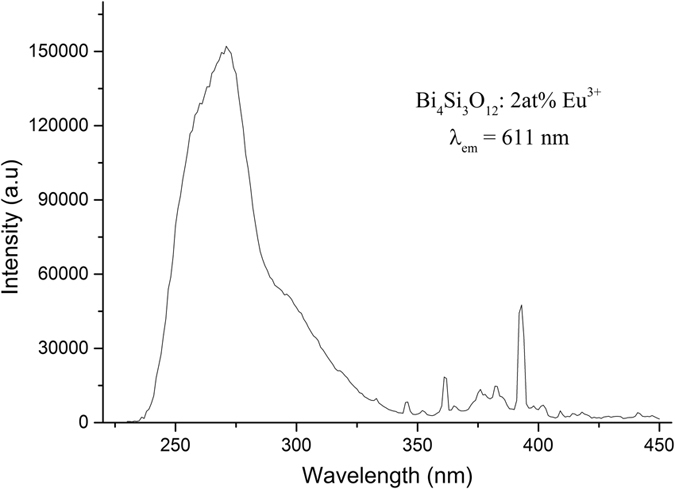
Excitation spectra of Bi_4_Si_3_O_12_: 2 at% Eu^3+^ phosphors at room temperature.

The excitation spectrum of 2at% Eu^3+^-doped Bi_4_Si_3_O_12_ phosphor is recorded by monitoring the emission at 611 nm corresponding to the ^5^*D*_0_–^7^*F*_2_ transition of Eu^3+^ (see Fig. 6). It consists of a broad absorption band along with a series of sharp peaks beyond 350 nm. The broad absorption band in 220–350 nm region (centered at 297 nm and 271 nm) is attributed to the transitions of 6 *s*^2^ → 6*s*6*p* of Bi^3+^ ions, and Eu^3+^ charge transfer band (CTB), which is due to charge transfer from the filled 2*p* orbital of the O^2−^ to the partially filled 4*f* orbital of Eu^3+^. It is well known that the ground state of Bi^3+^ ion with 6*s*^2^ configuration is ^1^*S*_0_ level. The 6*s*6*p* configuration of Bi^3+^ ion yields the^3^*P*_0_, ^3^*P*_1_, ^3^*P*_2_ and ^1^*P*_1_ excited states. In view of the spin selection rule and spin-orbit coupling, ^1^*S*_0_ → ^1^*P*_1_ and ^1^*S*_0_ → ^3^*P*_1_ transitions are expected^43^. The ^1^*S*_0_ → ^3^*P*_0_ and ^1^*S*_0_ → ^3^*P*_2_ transitions remain forbidden if configuration interaction is not taken into account^44^. However, the number and the position of Bi^3+^ absorption bands are sensitive to the coordination polyhedra offered by the host lattice^45^. It was reported that in pure Bi_4_Si_3_O_12_, the Bi^3+^ absorption band is at 285 nm^32^. An increase in covalency of Bi^3+^- O band in Bi_4_Si_3_O_12_: Eu^3+^ shifts this transition to lower energy due to the nephelauxetic effect^46^. Thus, the broad absorption band at 297 nm observed in the Bi_4_Si_3_O_12_: Eu^3+^ phosphors are assigned to the transition ^1^*S*_0_ → ^3^*P*_1_. In comparison with pure BSO samples, the ^1^*S*_0_ → ^3^*P*_1_ transition band for Bi_4_Si_3_O_12_: Eu^3+^ becomes broader and the absorption edge moves from 300 to 350 nm. The similar phenomena can be found in silicates, e.g., LiYSiO_4_^ ^47, Na_3_YSi_3_O_9_^ ^48, where only the ^1^*S*_0_ → ^3^*P*_1_ transition are observed. The other broad band with maximum at 271 nm corresponds to charge transfer (CT) transition within the [O^2−^ → Eu^3+^] complex. The excitation spectra of Eu^3+^ exhibit weak and narrow peaks caused by the direct excitation of the Eu^3+^ ground state into various higher levels of the 4*f* manifold. The sharp peaks centered at about 362, 383, 393, 413 nm are ascribed to the intra-configurational 4*f*–4*f* transitions in Eu^3+^ ion, which are ^7^*F*_0_ → ^5^*D*_4_, ^7^*F*_0_ → ^5^*L*_7_, ^7^*F*_0_ → ^5^*L*_6_, and ^7^*F*_0_ → ^5^*D*_3_ transitions of Eu^3+^, respectively. Though parity-forbidden *f*–*f* transitions of Eu^3+^ ions result in low absorption efficiency in the NUV and blue light, which would lead to potentially severe back scattering losses in LED packages and LED die, it may be improved by extra absorption involving the Bi–O component and CT band to broaden the absorption band. It makes the phosphor absorb NUV light efficiently and matches well with the NUV output wavelength of commercial LED chip.

**Figure 7:**
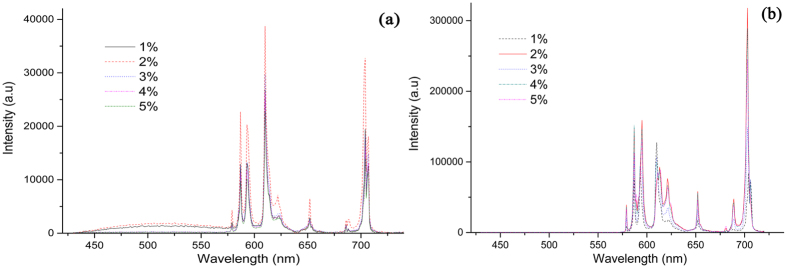
Emission spectra of Bi_4_Si_3_O_12_: *x*Eu^3+^ (*x* = 1 at%, 2 at%, 3 at%, 4 at%, 5 at%) phosphors under 271 nm (**a**) and 393 nm (**b**) excitation at room temperature

The emission spectra of the Bi_4_Si_3_O_12_: *x*Eu^3+^ (*x* = 1, 2, 3, 4, 5%) under excitation at 271 nm (corresponding to CTB of Eu^3+^ ion) and 393 nm excitation (corresponding to the *f*–*f* transition of Eu^3+^ ion) were presented in Fig. 7 at room temperature. Both emission spectra show characteristic emission of Eu^3+^. corresponding to the transitions starting from the excited state ^5^*D*_0_ of Eu^3+^ to the lower levels ^7^*F*_*J*_ (*J* = 0–4). More interestingly, the Eu^3+^ doped Bi_4_Si_3_O_12_ shows different luminescence properties upon different excitation wavelengths in these two spectra. The emission line at 579 nm corresponds to the ^5^*D*_0_ → ^7^*F*_0_ transition, and the presence of a single emission line for this transition confirms the Eu^3+^ ions occupy single crystallographic site in the lattice^49^. From Fig. 7(a), it is observed that the Eu^3+^ doped Bi_4_Si_3_O_12_ presents the dominant strong orange magnetic dipole (MD) transition at 594 nm upon the excitation of CTB at 271 nm. Figure 7(b) shows quite different results, with intense red forced electric dipole (ED) transition upon the excitation of 393 nm. It is interesting to notice that the predominant emission (forced ED transition at 611 nm or MD transition at about 594 nm) depends on not only the site symmetry of the Eu^3+^ ions but also the excitation wavelength. The intensity of ^5^*D*_0_ → ^7^*F*_2_ ED transition is strongly influenced by the local structure and the site symmetry around the Eu^3+^ ion while the intensity of ^5^*D*_0_ → ^7^*F*_1_ magnetic dipole transition is nearly independent of the host environment. The only one site (16c) for cation is occupied by Bi^3+^ with *C*_1_ site symmetry based on crystallographic structure of Bi_4_Si_3_O_12_. And no impurity phase in synthesis sample was found according to the XRD analysis. Thus, the difference observed in the emission characteristics of Eu^3+^ in Bi_4_Si_3_O_12_ under different excitation wavelengths could be explained by the delocalization effect in the excited state that results in the change of original *C*_1_ symmetry of Bi^3+^ site into a new micro-distortion site^50^. The emission spectra excited 271 nm exhibit a broad emission band at about 520 nm, which can be attributed to the typical emission of Bi^3+^ ions in accordance with a previous study^51^. While as the excitation wavelength changes to 393 nm, this emission corresponding to Bi^3+^ ions is gone. Besides peak position, the peak intensity also tightly depend on the excitation wavelength. The relative intensity of the emission peak excited with 393 nm wavelength was almost 8.5 times stronger than the emission peak excited with CTB. It implies that energy transfer from Bi^3+^ to Eu^3+^ ions happened and Bi^3+^ ions plays a dual role of luminescence center and a sensitizer for Eu^3+^ ions.

### Luminescence decay analysis

**Figure 8:**
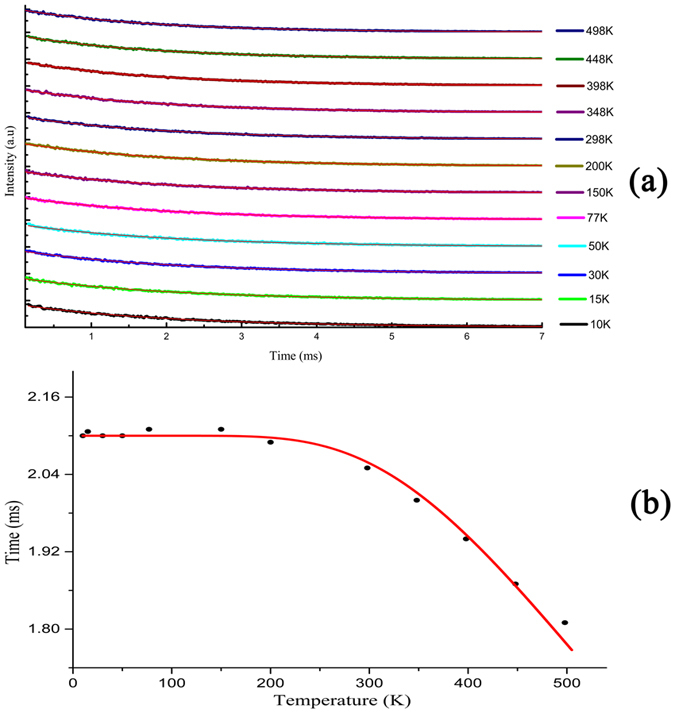
(**a**) Luminescence decay curves of Bi_4_Si_3_O_12_: 2 at% Eu^3+^ phosphor measured at 10–498 K by excitation at 393 m monitoring the ^5^D_0_–^7^F_2_ emission, (**b**) Luminescence decay lifetime as a function of temperature for Bi_4_Si_3_O_12_: 2 at% Eu^3+^. The dots are experimental data and the solid curve is the fit to the Eq. 2 in the text.

The temperature dependence of the decay profiles of 2 at% Eu^3+^ doped Bi_4_Si_3_O_12_ phosphor have been measured. The fluorescence decay curves corresponding to ^5^*D*_0_ → ^7^*F*_2_ transition around 611 nm upon 393 nm excitation were presented in Fig. 8(a). All decay curves measured can be described to good approximation to a single exponential function in the temperature 10–498 K, which confirms that Eu^3+^ occupy only one site. The lifetime value can be fitted as:


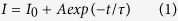


where *I*_0_ is the initial emission intensity for *t* = 0 and τ is the lifetime. The lifetime values are plotted in Fig. 8(b) as a function of recording temperature. As can been seen from Fig. 8(b), the lifetime is nearly temperature independent from 10 to 200 K and then it drops quickly with increasing temperature. The rapid drop of the Eu^3+^ emission lifetime for T > 200 K indicates the presence of a typical temperature quenching behavior between Eu^3+^ ions. In the thermal quenching process, energy transfer occurs from Eu^3+^ to killer centers or to any other defect centers near the Eu^3+^ ions by thermal phonon assistance.

Lifetime of the photoluminescence emission from the lowest excited state level can be defined as the inverse of the total radiative and nonradiative transition rates, and the non-radiative relaxation process is in competition with the radiative process. The temperature-dependent luminescence lifetime is fitted to the equation as:


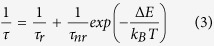


where Δ*E* is the energy gap between the energy levels, *k*_*B*_ is the Boltzmann constant, *1/τ*_*r*_ and *1/τ*_*nr*_ are the radiative and nonradiative decay rates, respectively. The probability for radiative process is assumed to be constant with temperature for a given material, whereas non-radiative transition is highly dependent on temperature. All relevant parameters are determined by least square fittings using a single exponential function. The fit to the experimental data according to Eq. (2) yields the following results: *τ*_*r*_ = 2.14 ms, *τ*_*nr*_ = 0.23 ms, and Δ*E* = 1769 cm^−1^ which is much higher than that of *β*–Ga_2_O_3_: Eu^3+^ (360 cm^−1^)^52^. Similar studied results are obtained from the temperature dependence of the time decay for Ca_2_Ge_7_O_16_: Eu^3+^ phosphors (1556 cm^−1^)^53^. According to Laporte selection rule for the electric-dipole (ED) *f*–*f* transitions, long luminescence decay is characteristic of the more symmetrical surrounding while short decay values are observed when site distortion occurs. The local symmetry around Eu^3+^ dopant is not centrosymmetric in *β*–Ga_2_O_3_: Eu^3+^, Ca_2_Ge_7_O_16_: Eu^3+^ and Bi_4_Si_3_O_12_: Eu^3+^ phosphors. However, Eu^3+^ occupied distorted octahedral site of Ga^3+^ ion in *β*–Ga_2_O_3_, and site distortion results in short lifetime and low activation energy^52^. Eu^3+^ ions substitute for Ca^2+^ ions easily in the lattice of Ca_2_Ge_7_O_16_ considering their similar radium, and co-doping Li^+^ ions can help to incorporate Eu^3+^ ions into Ca^2+^ sites and suppress the non-radiative processes. Thus, the values of lifetime and thermal activation energy of Bi_4_Si_3_O_12_: Eu^3+^ are close to the reported values in Eu^3+^-doped Ca_2_Ge_7_O_16_.

### Thermal quenching mechanism

**Figure 9:**
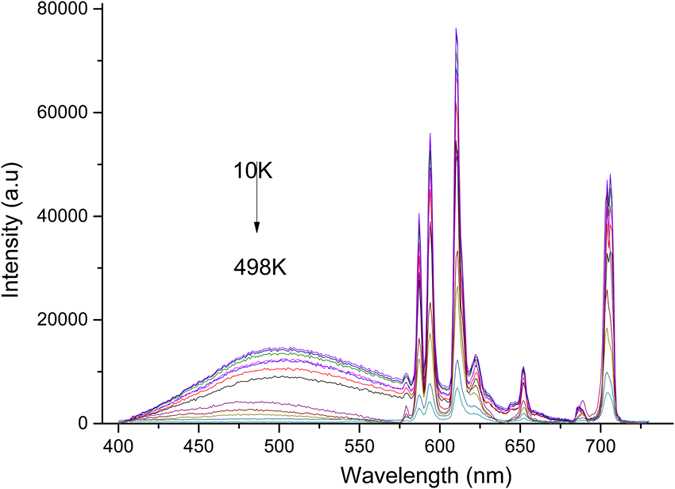
Emission spectra of Bi_4_Si_3_O_12_: 2 at% Eu^3+^ phosphors at different temperatures.

**Figure 10:**
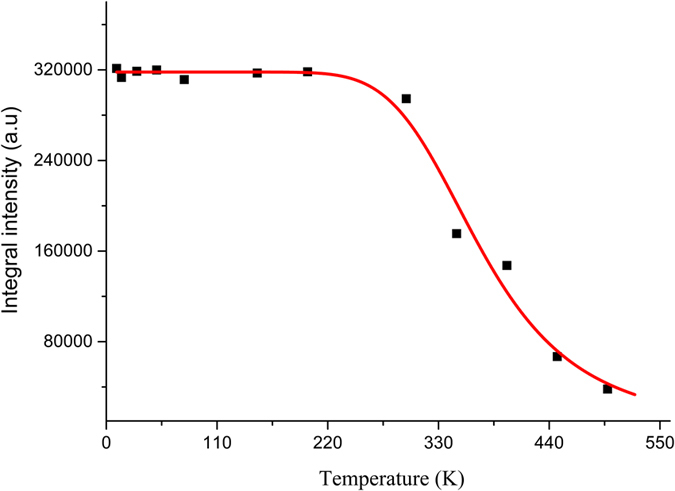
Temperature dependence of the integral emission intensity of 2 at% Eu^3+^-doped Bi_4_Si_3_O_12_ phosphors excited by 271 nm (squares) and the result of the fitting according to Eq. (4) **(solid line).**

Thermal stability of LED phosphor is one of the most important factors. For example, when high-power white LEDs operate, the temperature of the chip-phosphor package can reach above 423 K^54^. Thus, it is important to evaluate the thermal stability of phosphor for practical application. The emission spectra of 2 at% Bi_4_Si_3_O_12_: Eu^3+^ phosphors, measured at various temperatures ranging from 10 to 498 K and recorded at the excitation of 271 nm, are presented in Fig. 9. The positions of emission peaks at various temperatures remain almost unchanged, but temperature increase leads to the broadening of emission spectral bands. The broadening in spectral width and the decrease in emission intensity with increasing temperature can be described by thermal quenching^55^. With respect to the relationship between emission intensity for ^5^*D*_0_ → ^7^*F*_2_ and surrounding temperature, plotted in Fig. 10, the photoluminescence intensity increased gradually as the temperature declined. The thermal quenching temperature (*T*_*50*_) is defined as the temperature at which the emission intensity is 50% of its original intensity. Thus, the value of the thermal quenching temperature can be deduced from the intensity of emission peaks at different temperature in Fig. 9. The thermal quenching temperature *T*_*50*_ was found to be 398 K. When the temperature increases to 498 K, the luminescence intensity of the Bi_4_Si_3_O_12_: 2 at% Eu^3+^ phosphor is down to about 88% in comparison to that obtained at 298 K.

Thermal quenching effect referred to as temperature-dependent nonradiative processes. The typical nonradiative relaxation mechanisms of Eu^3+^ emission are (i) multiphonon relaxation, (ii) temperature dependent energy transfer, and (iii) crossover from the 4 *f*^6^ electronic configuration of Eu^3+^ ion to a charge transfer state. As can be seen from the Fig. 8(a), the fluorescence decay curves for the ^5^*D*_0_ state in 2 at% Eu^3+^-doped phosphor at various temperatures can be fitted well with either exactly or very nearly single exponential functions. The possibility that the observed temperature dependence of the nonradiative relaxation might be due to energy transfer to ligands or acceptor traps is doubted, since such a process result in a noticeable deviation from single exponential behavior^56^.

The temperature-dependent multiphonon relaxation process could be explained in the following manner^57^:


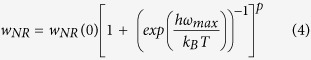


where *W*_*NR*_ is non-radiative decay rates, *W*_*NR*_(*0*) is the multiphonon decay rate at 0 K, *hω*_*max*_ represents the maximum phonon energy of all lattice vibrations in the molecule, *k*_*B*_ is the Boltzmann constant, and *T* is the temperature, *p* is the number of the phonons required to bridge the energy gap between the populated state and the adjacent low-lying state. The maximum phonon energy of the Bi_4_Si_3_O_12_ is about 991 cm^−1^, which is associated to the vibration mode of [SiO_4_] tetrahedral^58^. In view of the large energy gap (>16,000 cm^−1^) between the crystal field components of the emitting ^5^*D*_0_ level to the ground state ^7^*F*_2_ level, multiphonon relaxation mechanism seems to be not applicable in this case since more than 16 phonons are necessary to bridge the gap and cause effective quenching. Usually, phonons numbers no more than 6 can provide efficient relaxation in the weak coupling limit appropriate to the rare earth elements^59^. Therefore, it can be concluded that the energy transfer and the multiphonon relaxation are not the main reasons of the temperature quenching.

Having eliminated all other reasonable mechanistic pathways, we assert that the most probable thermal quenching mechanism involved nonradiation relaxation is most likely due to crossover process. The crossover quenching from the ^5^*D*_0_ excited state to a Franck-Condon shifted state is a thermal activation process. In crossover quenching mechanism, the temperature-dependent of the luminescent intensity can be described by the Struck-Fonger model equation^57^:


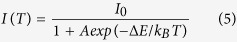


where *I*(*T*) is the luminescence intensity at a given temperature *T, I*_*0*_ is the initial luminescence intensity at the temperature of 0 K, *A* is a constant, *k*_*B*_ is Boltzmann constant, and Δ*E* is the activation energy for the thermal quenching process, which can be obtained by fitting the thermal quenching data. The experimental data for *f*–*f* integrated intensity, as shown in Fig. 10, are nonlinearly fitted by Eq. (5). The solid line in Fig. 10 shows the fitting curve. It can be found that the fitting curve matches well with the experimental data. The activation energy Δ*E* is obtained to be 0.24 eV for Bi_4_Si_3_O_12_: 2 at %Eu^3+^ phosphor according to a least-square fitting of the formula to the experimental data. The value of the activation energy Δ*E* is similar to those determined for other silicate based red phosphors, for example, NaCaGaSi_2_O_7_: Eu^3+^, Li^+^ (0.23 eV)^59^, Sr_2_Y_8_(SiO_4_)_6_O_2_: Bi^3+^/Eu^3+^ (0.23 eV)^60^, and CaAl_2_Si_2_O_8_: Eu^3+^ (0.27 eV)^61^. It illustrates good thermal stability of the obtained Bi_4_Si_3_O_12_: Eu^3+^ phosphor.

### CIE chromaticity coordinate and quantum efficiency of Bi_4_Si_3_O_12_: 2 at %Eu^3+^ phosphor

As is well known, photoluminescence spectrum for luminescent materials have characteristic temperature dependence, that is, with increasing of the temperature, the position of emission peak move to the lower energy region, the full width at half-maximum (FWHM) of emission band is broadened and the emission intensity is quenched at a certain temperature. These phenomena, thermal broadening, redshift of emission peak and the decrease in emission intensity, can be described by the interaction between the luminescence center and the vibrating crystalline environment^62^. As shown in Fig. 9, with the increase in the temperature from 10 up to 498 K, the emission intensities of Bi_4_Si_3_O_12_: 2 at% Eu^3+^ decrease, and the FWHM of Bi_4_Si_3_O_12_: 2 at% Eu^3+^ emission increases. However, positions of emission peak remain approximately constant with increasing temperature. The phonon interaction with the emitting center of Eu^3+^ ion is the dominating factor responsible for intensity quenching and the temperature broadening of Bi_4_Si_3_O_12_: Eu^3+^ luminescence spectral bands.

**Figure 11:**
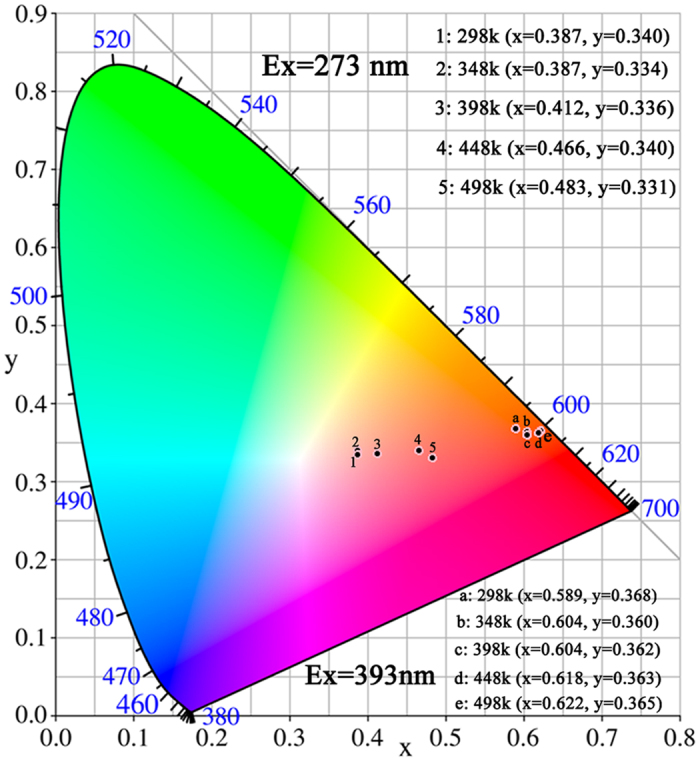
CIE chromaticity diagram of Bi_4_Si_3_O_12_: 2 at%Eu^3+^ excited by 271 nm and 393 nm at different temperatures.

To evaluate the influence of temperature on the chromaticity, the CIE chromaticity coordinates of Eu^3+^ doped Bi_4_Si_3_O_12_ phosphors are calculated according to the emission spectra for temperatures ranging from 298 to 498 K. The movement of the chromaticity point with temperature is shown in Fig. 11. Under excitation of 393 nm, the luminescent color tends to red light due to the increase of the relative intensity of the red component in currently synthesized phosphors. For comparison, the CIE color coordinates of the phosphor under the excitation of CTB are also provided. The CIE color coordinates of Bi_4_Si_3_O_12_: 2 at% Eu^3+^ vary from orange-red to red with the increase of temperature. Detailed CIE coordinate values upon 271 and 393 nm excitation are listed in Fig. 11. In addition, as evidenced by the excitation spectrum, the currently studied phosphor can effectively absorb the emissions from LED based NUV radiation. As the temperature increases, the interaction between luminescent centers and phonons holds dominant and, consequently the FWHM of the emission peak is broadened^63^. The spectra broadening leads to slight shift of the color point in the chromaticity diagram.

The quantum efficiency values of Bi_4_Si_3_O_12_: Eu^3+^ phosphor for Eu^3+^ red emission are determined to be 14.5% and 1.6% under excitation at 394 nm and 271 nm, respectively. This fact indicates that the energy absorbed by the CTB is transferred to Eu^3+^ ions levels nonradiatively and the non-radiative losses compared to radiative emission is relatively high in this phosphor under CT excitation. The quantum efficiency of Bi_4_Si_3_O_12_: Eu^3+^ phosphor under excitation at 394 nm is 14.5%, which is higher than that of commercial red phosphor Y_2_O_3_: Eu^3+^ (9.6%, 394 nm excitation)^64^, Y_2_O_2_S: Eu^3+^ (4.2%, 395 nm excitation)^65^. However, the quantum efficiency of Bi_4_Si_3_O_12_: Eu^3+^ is lower than that of the red-emitting nitride phosphor Sr_2_Si_5_N_8_: Eu^2+^ (75–80%, 465 nm excitation)^66^. Quantum efficiency of phosphor is often adopted as an important parameter in evaluating its potential application for solid state lighting. The quantum efficiency of Bi_4_Si_3_O_12_: Eu^3+^ phosphor can be further improved by optimizing synthetic procedure to reduce the surface defect, to get higher crystallization and to control gain morphology and size of phosphor.

## Methods

Commercially available high purity reagents (99.99%) of Bi_2_O_3_, SiO_2_ and Eu_2_O_3_ were used as raw materials to prepare Bi_4_Si_3_O_12_: xEu^3+^ phosphors (x = 1 at%, 2 at%, 3 at%, 4 at%,5 at%). The powders were weighed according to the chemical formula and ground and mixed together in a ball mill with an agate ball using an agate container for 8 h. After grinding and extruding to form pieces, the mixture was transferred to a small Al_2_O_3_ crucible that was in turn put into a larger covered Al_2_O_3_ crucible. The gap between two crucibles was filled with silicon carbide powder as the microwave susceptor. Considering the fact that the microwave penetration depth into silicon carbide layer decreases as the temperature rises, the amount of silicon carbide powder would be selected to ensure that the mixture was adequately received microwave irradiation at a certain temperatures. In order to minimize heat loss, the large crucible was placed in a cavity surrounded by the alumina insulation foam. The temperature was measured by inserting an infrared pyrometer into a small hole at the center of the inner crucible. Finally, the whole setup was placed into in a microwave heater (Synotherm Corp., HAMiLab-V3, China) with a rotating plate and a continuously adjustable output power from 0.2 to 2.8 kW at 650–800 °C for 10–30 minutes in air atmosphere.

The prepared samples were characterized by a Dmax 2500 X-ray diffractometer with CuKα radiation (Rigaku, Japan), with a scanning rate of 5°/min for phase identification and a step-scanning mode of 8 s per step (step size: 0.02°) for Rietveld analysis in the 2*θ* range from 12° to 90°. The structure refinement was performed using the Rietveld method with the General Structure Analysis System (GSAS) program and its graphical user interface, EXPGUI^67^,^68^. The grain size of the sample was analyzed by a Mastersizer 2000 laser scattering particle size analyzer (Malvern, UK). The morphology of the Eu^3+^ -doped Bi_4_Si_3_O_12_ phosphor was characterized using scanning electronic microscope (SEM FEI Quanta 200 FEG, USA). The photoluminescence excitation and emission spectra were recorded using Edinburgh Instruments FLS920 spectrophotometer and a 450 W Xenon lamp was used as an excitation source (EI, UK). Sample cooling was provided by a closed cycle liquid helium optical cryostat which allowed the temperature to be varied between 10 and 650 K (ARS, USA). The fluorescence quantum efficiency measurement by an absolute method was performed using the EI spectrophotometer equipped with an integrating sphere. The sphere with 120 mm diameter spherical cavity was coated with BENFLEC. The quantum efficiency were corrected by a series of direct and indirect measurements to remove the re-excitation of the sample from excitation light reflected within the sphere.

## Conclusion

Bi_4_Si_3_O_12_: Eu^3+^ phosphors have been successfully synthesized by a novel, fast and energy-efficient microwave irradiation method. The roughly spherical Bi_4_Si_3_O_12_: Eu^3+^ particles with few aggregation or agglomeration have been obtained by this route. The phase analysis and crystal structure of the phosphors were examined by XRD patterns and Rietveld refinement. The prepared samples exhibited single-phase with a cubic structure. The excitation spectrum consists of a broad band ranged from 220 nm to 350 nm, which assigned to the Eu^3+^ charge transfer transition and 6 *s*^2^ → 6*s*6*p* transition of Bi^3+^. and a series of sharp lines corresponding to the intra-configurational 4*f*–4 *f* transitions of Eu^3+^. Characteristic for the Eu^3+^ electric-dipole emission with the maximum at 610 nm was recorded. The luminescence decay of Bi_4_Si_3_O_12_: Eu^3+^ gradually decreases with increasing temperature, due to the energy transfer among Eu^3+^ ions occurring in a non-radiative process. With increasing of temperature the emission peaks show increasing bandwidth and decreasing intensity, which is due to the electron-phonon interaction with the luminescence center. The Bi_4_Si_3_O_12_: Eu^3+^ phosphor shows a typical thermal quenching effect with a *T*_*50*_ value of 398 K, and the thermal activation energy for the crossover process is calculated to be 0.24 eV. We have found that for the thermal quenching of Eu^3+^ luminescence the crossover relaxation mechanism is mainly responsible. In view of its strong and broad absorption band in the NUV region, intense red light with the appropriate chromaticity coordinate, this phosphor is competitive as a promising candidate for NUV LEDs.

## Additional Information

**How to cite this article**: Zhang, Y. *et al*. Eu^3+^-doped Bi_4_Si_3_O_12_ red phosphor for solid state lighting: microwave synthesis, characterization, photoluminescence properties and thermal quenching mechanisms. *Sci. Rep.* **7**, 42464; doi: 10.1038/srep42464 (2017).

**Publisher's note:** Springer Nature remains neutral with regard to jurisdictional claims in published maps and institutional affiliations.

## Figures and Tables

**Table 1:** Crystallographic data for (Bi_0.98_Eu_0.02_)_4_Si_3_O_12_ determined from Rietveld refinement.

Formular	(Bi_**0.98**_Eu_**0.02**_)_4_Si_3_O_12_			
Crystal system	Cubic			
Space group	I -4 3 d			
Cell parameters	*a* = *b* = *c* = 10.2831 Å			
	*α* = *β* = *γ* = 90°			
Cell Volume	1087.3625 Å^3^			
*Z*	4			
*R*_*wp*_	9.84%			
*R*_*p*_	7.10%			
*χ*^2^	4.17			
**Atom**	**Wyckoff position**	**Atoms coordinates**		
		***x***	***y***	***z***
Bi	16c	0.08503	0.08503	0.08503
Si	12a	0.37500	0.00000	0.25000
O	48e	0.06294	0.13624	0.29070
